# Observation of a topologically non-trivial surface state in half-Heusler PtLuSb (001) thin films

**DOI:** 10.1038/ncomms11993

**Published:** 2016-06-27

**Authors:** J. A. Logan, S. J. Patel, S. D. Harrington, C. M. Polley, B. D. Schultz, T. Balasubramanian, A. Janotti, A. Mikkelsen, C. J. Palmstrøm

**Affiliations:** 1 Materials Department, University of California-Santa Barbara, Santa Barbara, California 93106, USA; 2 MAX IV Laboratory, Lund University, Lund 221 00, Sweden; 3 Department of Electrical and Computer Engineering, University of California-Santa Barbara, Santa Barbara, California 93106, USA; 4 Department of Materials Science and Engineering, University of Delaware, Newark, Delaware 19716, USA; 5 Department of Physics, Lund University, Lund 221 00, Sweden

## Abstract

The discovery of topological insulators, materials with bulk band gaps and protected cross-gap surface states in compounds such as Bi_2_Se_3_, has generated much interest in identifying topological surface states (TSSs) in other classes of materials. In particular, recent theoretical calculations suggest that TSSs may be found in half-Heusler ternary compounds. If experimentally realizable, this would provide a materials platform for entirely new heterostructure spintronic devices that make use of the structurally identical but electronically varied nature of Heusler compounds. Here we show the presence of a TSS in epitaxially grown thin films of the half-Heusler compound PtLuSb. Spin- and angle-resolved photoemission spectroscopy, complemented by theoretical calculations, reveals a surface state with linear dispersion and a helical tangential spin texture consistent with previous predictions. This experimental verification of topological behaviour is a significant step forward in establishing half-Heusler compounds as a viable material system for future spintronic devices.

## Abstract (web summary)

Half-Heusler compounds are predicted to bear topological properties, which is yet to be experimentally evidenced. Here, Logan *et al.* report experimental evidence of a topological surface state in epitaxially grown thin films of the half-Heusler compound PtLuSb, providing a candidate for new spintronic devices.

## Graphical abstract

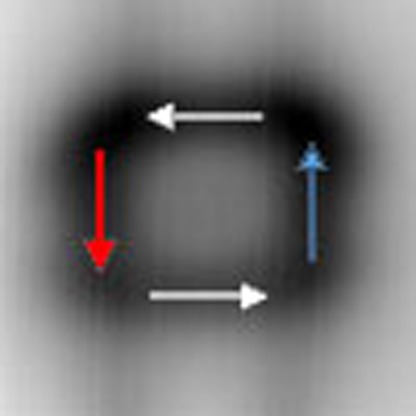

Topological insulators are a recently discovered new state of matter, which possess bulk band gaps and metallic surface states. Their experimental realization in materials such as Bi_2_Se_3_ (ref. 1) has spurred a number of efforts to observe new physics and computationally predict other host compounds^2^,^3^. One particularly promising avenue for a new family of topologically non-trivial materials is the half-Heusler family. Half-Heusler compounds are ternary intermetallics (*XYZ*), which share strong structural similarities to III–V zinc blende binary semiconductors. Crystallographically, these compounds can be thought of as a zinc blende lattice of the *X* and *Z* atoms, with additional *Y* atoms introduced in the octahedral sites. Depending on the particular elemental species involved, half-Heusler compounds have a much wider range of electronic structures than III–Vs, owing to the important role that the total valence electron count per formula unit has on the Fermi level position^4^. For instance, Heusler compounds have previously been shown to exhibit electronic behaviours such as half-metallic ferromagnetism^5^, superconductivity^6^ and semiconductivity^7^. Consequently, this flexibility allows for the design of heterostructures of the same crystal structure, but highly varying electronic and magnetic properties. In addition, this enables the possibility of discovering materials with combined properties, such as topological superconductors, which are of interest for the appearance of Majorana fermions^3^,^8^.

For this study, we examine the electronic structure of the half-Heusler compound PtLuSb (001). Samples were grown by molecular beam epitaxy and characterized by reflection high-energy electron diffraction and X-ray diffraction, to ensure high sample quality. First-principles calculations^9^,^10^,^11^ predict PtLuSb to lie at the border between normal and inverted band ordering with a zero-gap semiconducting band structure. Previous experimental studies on bulk single crystals^12^,^13^ and thin films^14^ have confirmed that PtLuSb has the expected zero-gap density of states, but have not measured the momentum-resolved electronic band structure. Consequently, we use angle-resolved photoemission spectroscopy (ARPES) to directly probe the electronic band structure and search for the predicted topological surface state (TSS). Critically, we also use spin resolution to discern the spin texture and ensure no spin-opposite partner bands are present. With this capability, we point-by-point identify all of the relevant signatures of a TSS: first, that no out-of-plane dispersion is observed, due to the confined surface nature; second, that a linear in-plane dispersion is apparent, with no evidence of partner bands; and third, that helical spin–momentum locking is evident^2^. Experimental ARPES data are directly compared with new *ab-initio* calculations that further support the results.

## Results

### Theoretical PtLuSb electronic structure

**Figure 1:**
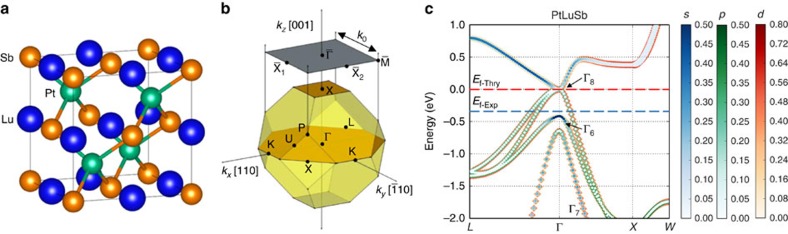
PtLuSb bulk crystallographic and electronic structure. (**a**) Half-Heusler C1_b_ crystal structure consisting of three interpenetrating face-centred-cubic sub-lattices. For PtLuSb, the platinum, lutetium and antimony atoms are denoted by the green, blue and orange spheres, respectively. (**b**) Half-Heusler bulk Brillouin zone with the (001) surface Brillouin zone projection. The high-symmetry surface Brillouin zone points are defined as 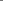
(0, 0), 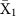 (*k*_0_/2, 0), 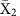 (0, *k*_0_/2) and 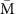 (*k*_0_/2, *k*_0_/2), where, for PtLuSb (001), the surface unit momentum *k*_0_=2*π*(√2/*a*_0_)=1.376 Å^−1^. (**c**) First-principles calculated bulk electronic band structure of PtLuSb with corresponding band character shown. The experimental Fermi level (blue), *E*_f-Exp_, is ∼0.35 eV below the calculated Fermi level (red), *E*_f-Thry_. A band inversion can be clearly seen at the bulk Γ point, where the orbital character of the Γ_8_ and Γ_6_ bands are inverted. This is in agreement with prior predictions^11^ that the TSS should connect these two bands.

Recent first-principles calculations have suggested that numerous half-Heusler compounds may exhibit topologically non-trivial behaviour^9^,^10^,^11^,^15^,^16^. Focusing on the high average atomic number 18 valence electron per formula unit topological insulator candidates, these materials are predicted to exhibit a zero-gap semiconducting or semimetallic bulk band structure, where, as a result of the interaction between the chemical bonding, crystal field splitting and spin–orbit coupling, an inversion between the Γ_8_ (*p*-character) and Γ_6_ (*s*-character) bands occurs across the Fermi level^9^,^10^,^11^. This band inversion is further predicted to induce the formation of a TSS with linear dispersion, spin–momentum locking and a Dirac-like crossing similar to those seen in Bi_2_Se_3_ (ref. 1). Examining the *ab-initio*-calculated bulk band structure for PtLuSb (Fig. 1c), we also observe that, unusually, owing to the position of the lower Γ_8_ band, the TSS should exist in a region where it threads between inverted bulk bands despite the presence of an extra bulk band (similar to that in HgTe^17^ and α-Sn^18^,^19^).

### In-plane ARPES

**Figure 2:**
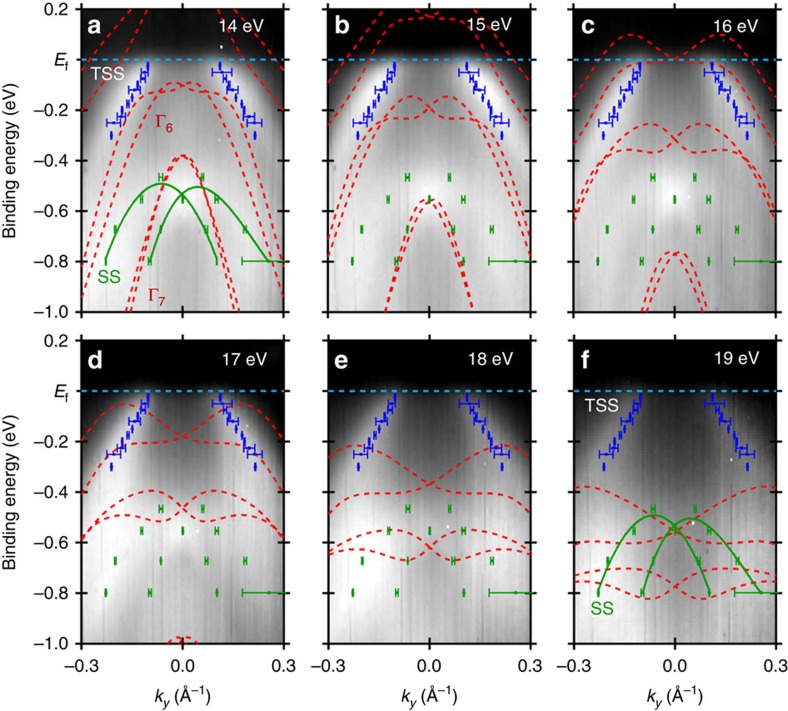
Experimental and calculated in-plane band structure. In-plane constant photon energy ARPES dispersion maps and corresponding constant *k*_*z*_ calculated bulk band structure projections (red) with overlaid extracted surface state positions (blue and green) along the 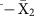 direction for incident photon energies of (**a**–**f**) 14–19 eV, respectively. The maps highlight the presence of two surfaces states, which do not disperse as a function of changing *k*_*z*_, unlike the bulk bands. The theory calculation Fermi level has been shifted −0.35 eV to align with the experimental Fermi level position. Error bars correspond to 1 s.d. for Voigt peak fits.

To begin experimentally, we examine the in-plane ARPES dispersion maps for various photon energies (Fig. 2), to perceive the bulk band motion relative to any surface states. Changing the incident photon energy results in an adjustment in *k*_z_ enabling bulk bands, which disperse with *k*_z_, to be easily distinguished from surface states, which do not disperse in *k*_z_, at normal emission (*k*_x_, *k*_y_=0). For PtLuSb (001), an incident photon energy of 13 eV corresponds to the bulk Γ point at the Fermi level, whereas an incident photon energy of 26 eV corresponds to the bulk *X* point at the Fermi level, based on the bulk band periodicity. By comparing spectra taken at 14–19 eV, we observe two sets of high-intensity bands that are non-dispersive with photon energy (position extractions from multi-peak Voigt fits are overlaid on the surface projections of the calculated bulk bands—see Supplementary Note 1 for theory calculation alignment): one set that crosses the Fermi level (blue) and one set ∼0.5 eV below the Fermi level at 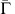 (green). In addition, we note the presence of several weaker intensity bands, which appear to move downward in binding energy as *k*_z_ moves towards the bulk *X* point (higher photon energies), consistent with the projected bulk bands (red). The low intensity of these bulk bands is a combination of the extremely high intensity of the non-dispersive states and the relative sensitivity of the incident linearly polarized light to bands with different character. Here, the lower bulk Γ_8_ and Γ_6_ (away from 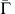) have mixed *p*_*y*_ and *d* character, whereas the experimental setup is more sensitive to *p*_*x*_ and *p*_*z*_ character. Examining these non-dispersive states more closely, which are not explained by our first-principle bulk band calculations (suggesting a surface origin), we first note the lower state's similarity to a Rashba-like split hole band (see Supplementary Note 2 for detailed analysis). This split hole band is reminiscent, both in terms of shape and binding energy, to features seen in ARPES measurements of cleaved bulk PtLuBi (111) and PtGdBi (111) half-Heusler crystals^20^. Consequently, it is reasonable to assume that this lower surface state arises due to a common crystallographic or chemical feature shared by a variety of high Z half-Heusler compounds. Second, we note that the upper non-dispersive state is qualitatively similar to the lower half of a Dirac cone, matching with the expectation for a TSS.

### Surface-state identification

**Figure 3:**
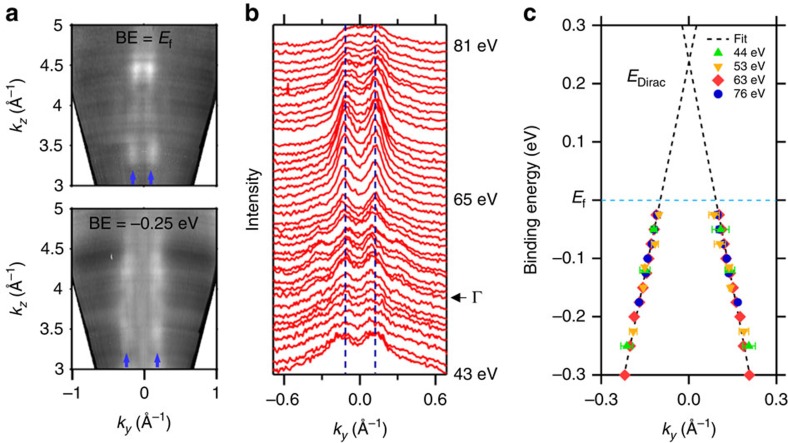
Out-of-plane-momentum *k*_*z*_ dependence and surface state extrapolation. (**a**) Intensity as a function of out-of-plane and in-plane momenta for various binding energy slices. An absence of *k*_*z*_ dispersion can be observed, consistent with the behaviour of a surface state. (**b**) Corresponding waterfall plot at a binding energy of −0.075 eV for photon energies between 43 eV (*k*_*z*_ ≈ 3.65 Å^−1^) and 81 eV (*k*_*z*_ ≈ 4.82 Å^−1^). Examining the spectra closely, an intensity increase can be seen near 65 eV (approximately one-third of the way from the bulk Γ point towards the bulk *X* point). This agrees with the calculated bulk band motion as the lower Γ_8_ band crosses the constant binding energy surface. (**c**) By tracking the surface-state position through binding energy for various photon energies, the predicted linear in-plane dispersion can be seen. The fitted peak positions are shown for several photon energies and no significant difference is observed. The Dirac point can be extrapolated to ∼0.24 eV above the Fermi level. Error bars correspond to 1 s.d. for Voigt peak fits.

To prove that this observed Dirac-like state arises from the sample's surface, we scan the incident photon energy over a wide range at normal emission (corresponding to sweeping along bulk Γ–*X* for several Brillouin zones) and simultaneously examine the binding energy dependence along the 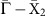 direction (Fig. 3a). We immediately note a lack of *k*_z_ dispersion, in agreement with our initial observations from the photon energy varying in-plane snapshots (Fig. 2) and consistent with the behaviour of a surface state. By examining a waterfall plot for a range of photon energies at a constant binding energy (Fig. 3b), we notice that intensity modulations appear within the surface state, which closely correspond with the values of the bulk Γ points^21^, and momenta where the calculated bulk bands cross the constant binding energy surface. Furthermore, by extracting the surface-state position for various binding energies and photon energies, a linear dispersion can be seen (Fig. 3c). Extrapolating this Dirac-like dispersion upward yields a crossing ∼0.24 eV above the Fermi level (see Supplementary Note 3 for additional detail). The extraction suggests the TSS may exist in an energy range on the order of ∼1 eV, larger than expected. This may be the result of the most negative data points occurring after the TSS merges with the bulk or due to underprediction in the theory. Furthermore, in agreement with a previous experimental study^14^ where Hall measurements of similar films revealed ∼1 × 10^20^ cm^−3^ *p*-type carriers, we observe that the Fermi level falls within the valence band. This is in contrast with theory calculations, which predict the Fermi level to fall at the valence band maximum, suggesting that there may be a low-energy defect that induces *p*-type doping, similar to antimony antisite disorder in III-Sb semiconductors^22^.

### Fermi-surface mapping

**Figure 4:**
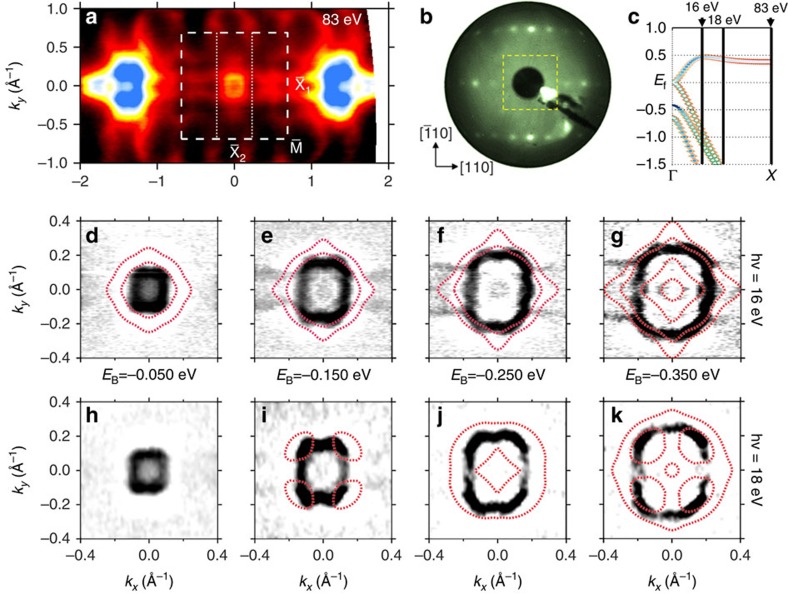
Experimental and calculated FS maps. (**a**) The observed constant binding energy surface of PtLuSb (001) with an incident photon energy of 83 eV (the bulk *X* point) and binding energy of 0.075 eV. The higher photon energy increases the amount of accessible reciprocal space at a cost of resolution. A threefold periodic state can be seen in addition to the TSS. The second-order 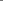 points (*k*_*x*_=±*k*_0_, *k*_*y*_=0) have greatly increased intensity, because they project from near the bulk Γ point of the adjacent Brillouin zone enabling both surface and bulk states to be seen. (**b**) Low-energy electron diffraction pattern (75 eV) of the sample's surface revealing a (1 × 3) surface reconstruction. (**c**) Schematic diagram highlighting *k*_*z*_ position of the 16, 18 and 83 eV FS maps. Finally, high-resolution second-derivative FS maps near 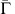 for an incident photon energy of (**d**–**g**) 16 and (**h**–**k**) 18 eV for various binding energies showing a complex Fermi-surface shape, overlaid with the calculated bulk structure. The theory calculation Fermi level has been shifted −0.35 eV to align with the experimental Fermi level position.

To check for other surface states and obtain a more complete picture of the Fermi surface (FS), a higher photon energy can be used to increase the accessible reciprocal space area. As seen in Fig. 4a, it is clear that the FS is more complicated than existing calculations suggest. The sample's (1 × 3) surface reconstruction, seen in low-energy electron diffraction (Fig. 4b), contributes an additional replica state with a threefold periodicity in the 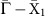 direction. The intensity difference between the first and second 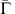 points is consistent with considerations of the bulk Brillouin zone projections (see Supplementary Fig. 4). At this photon energy, the first 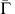 point projects from near the bulk *X* point, where the bulk bands are far from the Fermi level, whereas the second 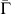 point projects from near the bulk Γ point of an adjacent Brillouin zone, leading to overlap of surface and bulk bands, and greatly increased intensity. Focusing on the potential TSS near 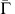 more closely, we measure constant energy contours at photon energies of 16 eV (Fig. 4d–g) and 18 eV (Fig. 4h–k). Similar to that in Bi_2_Te_3_ (111)^23^,^24^ and W (110)^25^, where warping is seen, the constant energy contours are anisotropic, eventually resembling the square of the (001) surface Brillouin zone at binding energies far from the Dirac point. Furthermore, the linear state's rectangular FS is convoluted with features from the threefold state leading to the appearance of distortions, in particular along the shorter edge at lower binding energies. By examining the second-derivative maps we note the presence of several weak features closer to 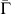 than the surface state with linear dispersion, which disappear as photon energy is increased to 18 eV. Consequently, we attribute these states to the bulk bands, which agrees well with our calculated bulk FS structure.

### Spin-texture identification

**Figure 5:**
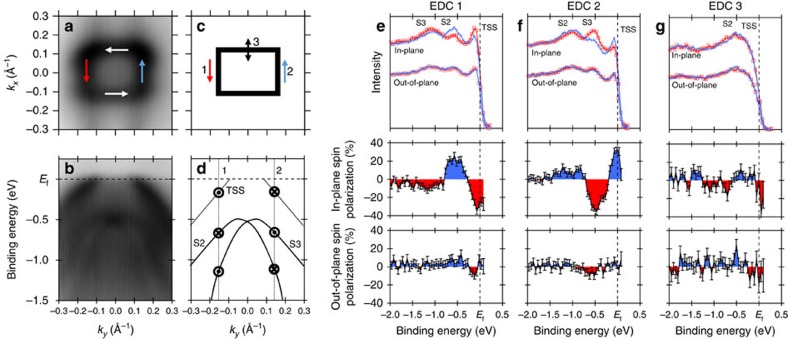
Spin texture of the topologically non-trivial surface state. (**a**) Constant binding energy surface with a photon energy of 16 eV highlighting the surface state with linear dispersion. (**b**) In-plane ARPES dispersion along the 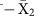 direction emphasizing the position of the surface state with linear dispersion and the lower Rashba-like surface state. (**c**) Schematic FS diagram depicting the location of energy distribution curves (EDCs) 1–3. (**d**) Schematic in-plane ARPES dispersion with the measured in-plane spin-polarization marked. The surface state with linear dispersion shows strongly opposing polarizations on either side. Furthermore, the spin polarization for Rashba-like surface state alternates as expected. (**e**–**g**) EDCs and spin polarizations for the noted locations. Measured intensities are shown for the in-plane and out-of-plane spin EDCs. For in-plane spin polarization, positive (blue) spin polarizations correspond to polarization towards positive *k*_*x*_, negative (red) spin polarizations correspond to polarization towards negative *k*_*x*_. For out-of-plane spin polarization, positive (blue) spin polarizations correspond to polarization towards positive *k*_*z*_, negative (red) spin polarizations correspond to polarization towards negative *k*_*z*_. Consequently, considering EDCs 1–3 we note the presence of a anticlockwise helical spin texture with minimal radial component. Error bars are correspond to the statistical error from counting statistics (further discussion in Supplementary Note 4).

Lastly, we conduct spin-ARPES to map the spin texture of the surface states closely surrounding 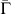 and then ensure no partner bands are present. By examining the spin polarization and energy distribution curves for various points on the FS (Fig. 5e–g), it becomes clear that the surface state with linear dispersion has a strong spin texture with either side having opposite polarization. Furthermore, we observe a anticlockwise helical tangential spin texture with minimal radial components, similar to that seen in the lower cone of Bi_2_Se_3_ (ref. 1) and consistent with theoretical calculations for a TSS in half-Heusler compounds with positive spin–orbit coupling^15^. We also observe that the surface state at lower binding energy is spin split, consistent with the expectation for a Rashba-like split hole band. In both cases, although our calculations suggest the bulk bands may also exhibit spin splitting (see Supplementary Fig. 5 for more detail) due to the lack of bulk inversion symmetry, the low detection efficiency for spin-ARPES causes only the highest intensity states to be measured. Consequently, when we consider the intensity difference seen between the bulk and surface states in Fig. 2, the observed spin signal must be dominated by surface state behaviour (see Supplementary Note 4 for additional details on the polarization calculations).

## Discussion

Although the experimental data are well explained by a TSS, a possible alternative explanation is that the upper surface state is instead a trivial surface state with unequal contributions of bulk (Dresselhaus) and structural (Rashba) inversion asymmetries, similar to that shown by Ganichev and Prettl^26^. However, several observations argue against such an interpretation. First, a Rashba/Dresselhaus combination state requires the presence of a partner band with opposite spin polarization. Examining our spin-ARPES data, we do not observe any such behaviour. Furthermore, in the event that partner bands were too close together to resolve in momentum space, the observed spin polarization would be very weak or non-existent, in contradiction with the measured data. Second, examining the spin-integrated ARPES data, we note that although there is some residual intensity between the Fermi level and the lower surface state with an incident photon energy of 16 eV, the intensity drops rapidly as *k*_*z*_ is moved towards the bulk *X* point, consistent with the bulk band motion. Third, the surface-state extraction fits to a linear dispersion with high accuracy. This is in contrast with the expectation for a trivial Rashba/Dresselhaus surface state, where a purely linear dispersion would not be expected^26^. Finally, the observed surface-state behaviour corresponds well, both with theory in literature as well as our own first-principles calculations. Therefore, we conclude that no partner bands are present, and that consequently the observed surface state is not a trivial Rashba/Dresselhaus surface state.

In summary, we report the direct experimental observation of a topologically non-trivial surface state in the half-Heusler material system. Our spin-ARPES data of PtLuSb (001), supported by first-principles calculations, reveal a topological state that has an anticlockwise helical spin polarization and extrapolated Dirac point ∼0.24 eV above the Fermi level. It is important to note that although unstrained PtLuSb is a zero-gap semiconductor, it demonstrates that half-Heusler compounds may exhibit topologically non-trivial surface states. With this experimental verification, it opens the door for the numerous other compounds in the Heusler family that have been suggested to possess the necessary bulk band inversion, some of which have non-zero bulk bandgaps. This continues to highlight the multifunctional nature of Heusler compounds by demonstrating the presence of another unique electronic structure, potentially enabling a range of new heterostructure devices that combine the widely varied electronic properties of these materials without changing crystal structure. Consequently, Heusler compounds are an exciting and promising direction for further exploration to find new topologically non-trivial materials and for the potential to discover materials with new electronic structures such as topological superconductors.

## Methods

### Growth approach

Samples consisting of 18 nm of relaxed PtLuSb on five monolayers of GdSb on relaxed ∼1 × 10^18^ cm^−3^ beryllium-doped Al_0.1_In_0.9_Sb on *p*+ GaAs (001) substrates with an ∼200-nm antimony cap were grown by molecular beam epitaxy with a system base pressure of <1 × 10^−10^ Torr. 500-nm thick, ∼1 × 10^18^ cm^−3^ beryllium-doped GaAs buffer layers were first grown under an As_4_ overpressure to form a smooth (2 × 4) surface reconstruction. Al_0.1_In_0.9_Sb was then nucleated directly on the GaAs at 380 °C by first soaking the GaAs surface with Sb_2_ for 10 s and then beginning co-deposition. Growth was continued as the substrate was quickly heated to 450 °C, to smooth the surface and prevent excess antimony from sticking, similar to the technique developed by Davis *et al.*^27^. Al_0.1_In_0.9_Sb layers were terminated by an asymmetric (1 × 3) surface reconstruction after 300 nm of growth. Five monolayers of GdSb, calibrated by Rutherford backscattering spectrometry of elemental films and reflection high-energy electron diffraction (RHEED) oscillations^28^, were then grown to provide a diffusion barrier^29^ between the PtLuSb and the Al_0.1_In_0.9_Sb buffer layer structure. Samples were then transferred *in situ* to a separate molecular beam epitaxy system for PtLuSb growth. Lutetium and antimony were evaporated from effusion cells, whereas platinum was evaporated from an electron beam evaporator. Beam fluxes were calibrated by a combination of Rutherford backscattering spectrometry and RHEED intensity oscillations for lutetium and antimony, and by a quartz crystal microbalance for platinum, to obtain a 1 lutetium:1 platinum:1.2 antimony atomic flux ratio. Nucleation of the PtLuSb was initiated with a low-temperature shuttered sequential monolayer growth technique followed by co-deposition at 380 °C using the procedure developed by Patel *et al.*^14^,^30^. At this temperature, antimony is self-limited, similar to the Al_0.1_In_0.9_Sb growth. RHEED patterns showed either streaky (1 × 3) or c(2 × 2) surface reconstructions, depending on antimony overpressure and substrate temperature. Finally, to prevent oxidation during *ex situ* transfer to the MAX-lab synchrotron facility, a ∼200 nm antimony capping layer was deposited at 100 °C (see Supplementary Fig. 1). X-ray diffraction analysis shows clear thickness fringes consistent with the expected growth rate.

### Experimental approach

ARPES and spin-ARPES measurements were taken at beamlines I4 and I3, respectively, at the MAX-lab synchrotron facility in Lund, Sweden. Before measurement, samples were heated to ∼390 °C whereupon the antimony capping layer was desorbed, as confirmed by low-energy electron diffraction and examination of shallow core levels by angle-integrated photoemission spectroscopy. Spin-integrated ARPES was conducted at 100 K, to reduce thermal broadening, whereas spin-resolved ARPES was conducted at 300 K with vacuum conditions of 1 × 10^−10^ Torr. The observed band structures were stable throughout the entire measurement duration.

### Theoretical approach

The calculations are based on the density functional theory within the generalized gradient approximation as implemented in the VASP code^31^. The interactions between the valence electrons and the ion cores were described using projected augmented wave potentials. A planewave basis set with cutoff of 300 eV was employed and a special *k*-point mesh of 12 × 12 × 12 was used for the face-centred cubic primitive cell. The calculated *a*_0*=*_6.512 Å is less than 1%, larger than the experimental value of 6.457 Å. A *k*-point mesh 8 × 8 × 6 was used in the calculations for the six atom tetragonal cell (√2/2*a*_0_ × √2/2*a*_0_ × *a*_0_), where *a*_0_ is the equilibrium lattice parameter. The electronic band structure calculations were performed including spin–orbit interaction.

### Data availability

The data that support the findings of this study are available from the corresponding author upon request.

## Additional information

**How to cite this article:** Logan, J. A. *et al.* Observation of a topologically non-trivial surface state in half-Heusler PtLuSb (001) thin films. *Nat. Commun.* 7:11993 doi: 10.1038/ncomms11993 (2016).

## Supplementary Material

Supplementary Information Supplementary Figures 1-6, Supplementary Notes 1-4 and Supplementary References
