# Supplementary material for: Observation of a topologically non-trivial surface state in half-Heusler PtLuSb (001) thin films
Source: Nat Commun. 2016 Jun 27;7:11993. doi: 10.1038/ncomms11993 (PMC4931221; doi:10.1038/ncomms11993)
Supplement: Supplementary Information — Supplementary Figures 1-6, Supplementary Notes 1-4 and Supplementary References [file ncomms11993-s1.pdf]

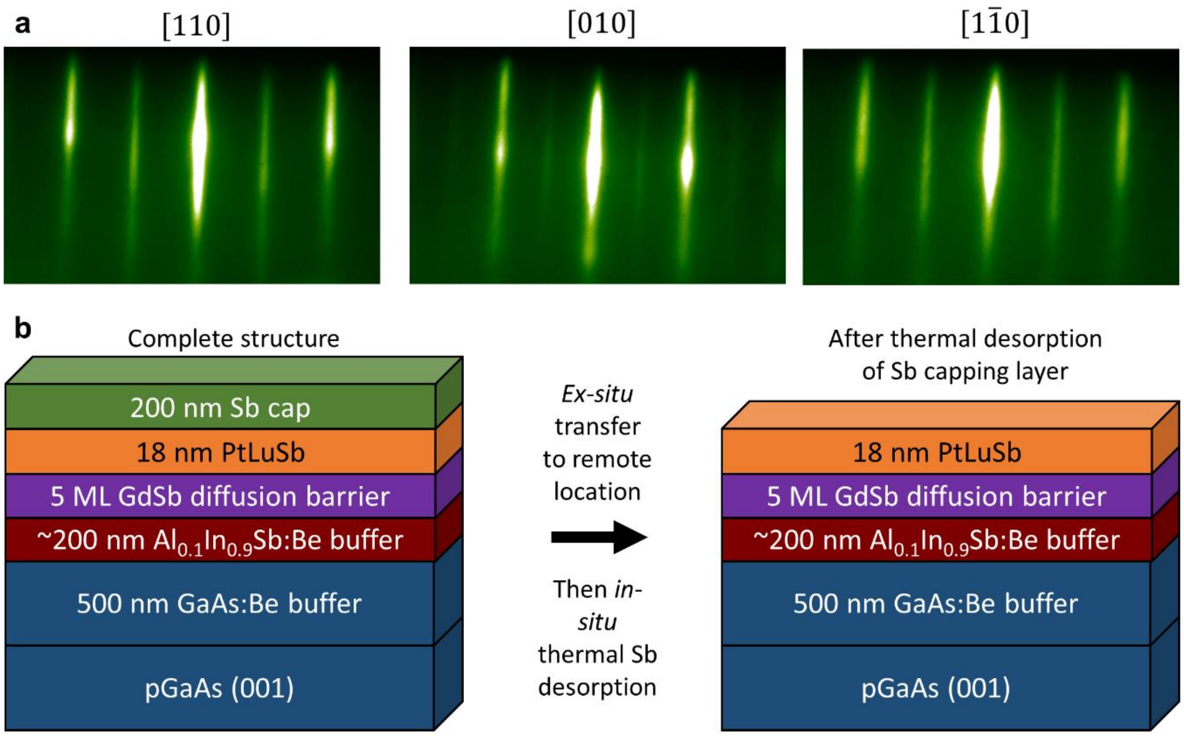

Supplementary Figure 1 | PtLuSb RHEED and sample structure before and after capping layer desorption. **a**, Reflection high-energy electron diffraction patterns of the 18 nm PtLuSb film prior to deposition of the protective antimony capping layer showing a sharp streaky c(2x2) pattern consistent with a smooth, high quality film. **b**, Schematic diagram of the sample structure before and after thermal desorption of the antimony capping layer. This capping layer prevents the PtLuSb from oxidizing during *ex-situ* transfer between the growth system and the ARPES system.

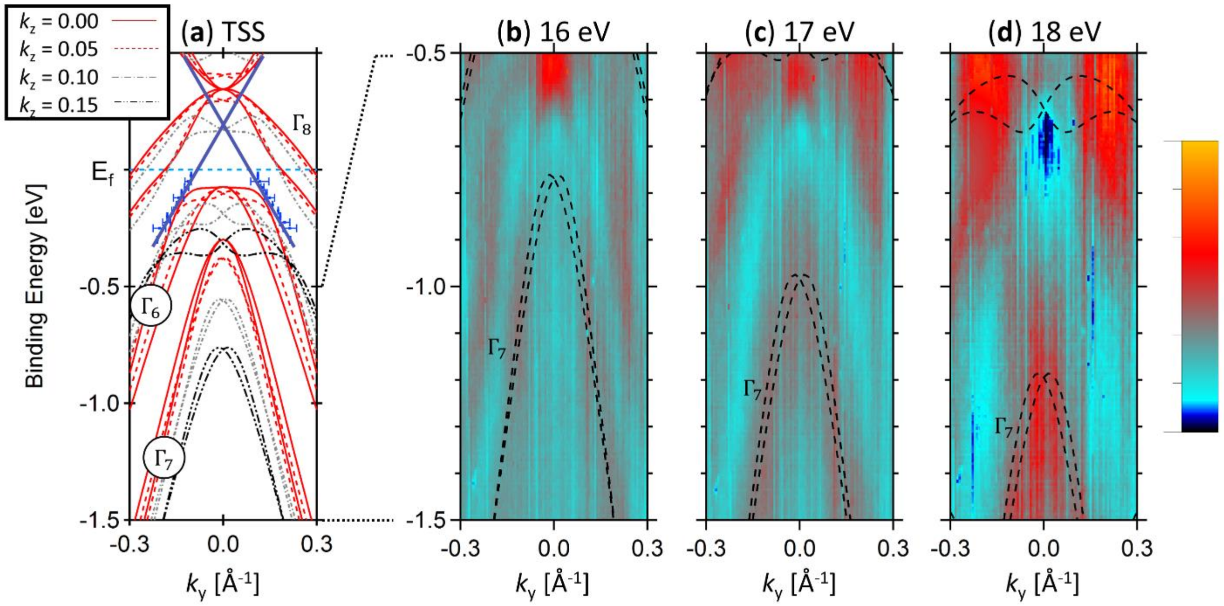

Supplementary Figure 2 | Theory calculation alignment. a, Combined overlay for various normalized k_z surface projections near the Γ point of the calculated bulk band structure with the extracted TSS position. Normalized k_z = 0 corresponds to the bulk Γ point and normalized k_z = 0.5 corresponds to the bulk X point. **b-d**, In-plane ARPES snapshots for an incident photon energy of 16 eV, 17 eV, and 18 eV, respectively. By applying a high pass filter and adjusting the color and contrast the bulk Γ_7 band motion can be seen. The theory calculation Fermi level has been shifted -0.35 eV to align with the experimental Fermi level position.

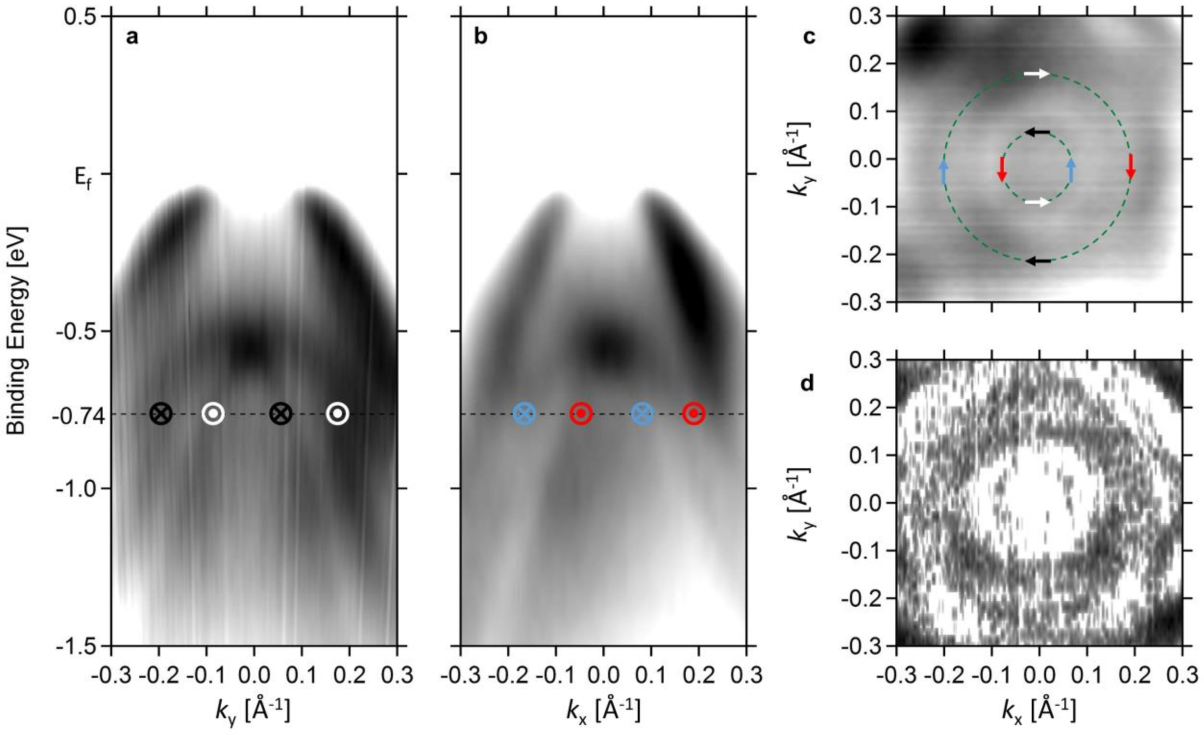

Supplementary Figure 3 | Rashba-like surface state analysis. In-plane ARPES snapshots for (**a**) $k_x = 0$ and (**b**) $k_y = 0$ with the measured spin direction marked. **c**, Constant energy surface at a binding energy of -0.74 eV; the dotted lines are guides to the eye for the position of the Rashba-like state and the arrows show the measured orientation of spin polarization from Fig. 5 in the main text. **d**, Second derivative analysis of (c) highlighting the surface state position.

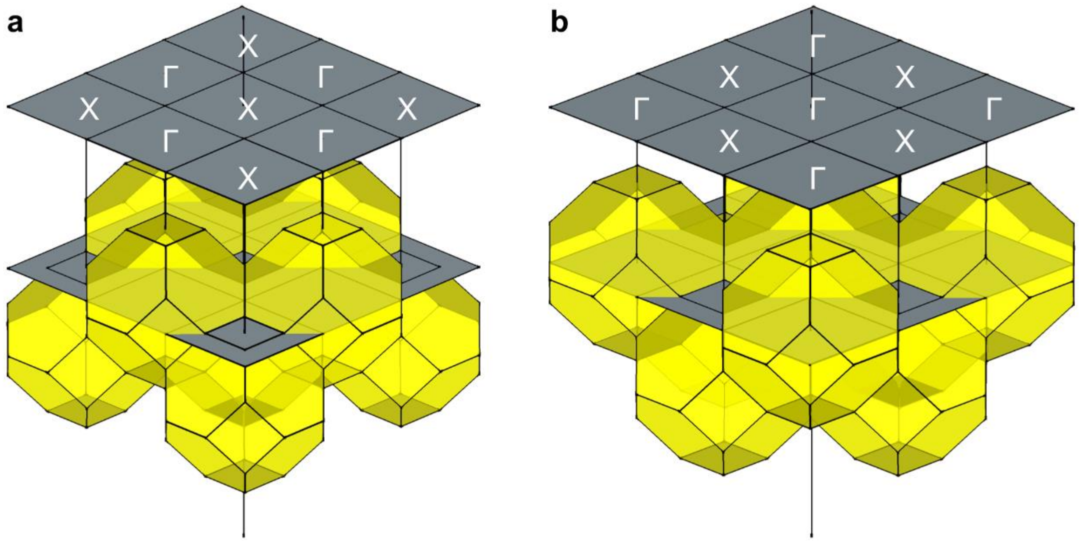

Supplementary Figure 4 | Expanded surface unit cell projections. Bulk to (001) surface Brillouin zone projections including neighboring bulk zones centered at (**a**) the bulk X point and (**b)** the bulk Γ point. Labels correspond to the bulk point projecting to $\bar{\Gamma}$ for the respective surface Brillouin zone.

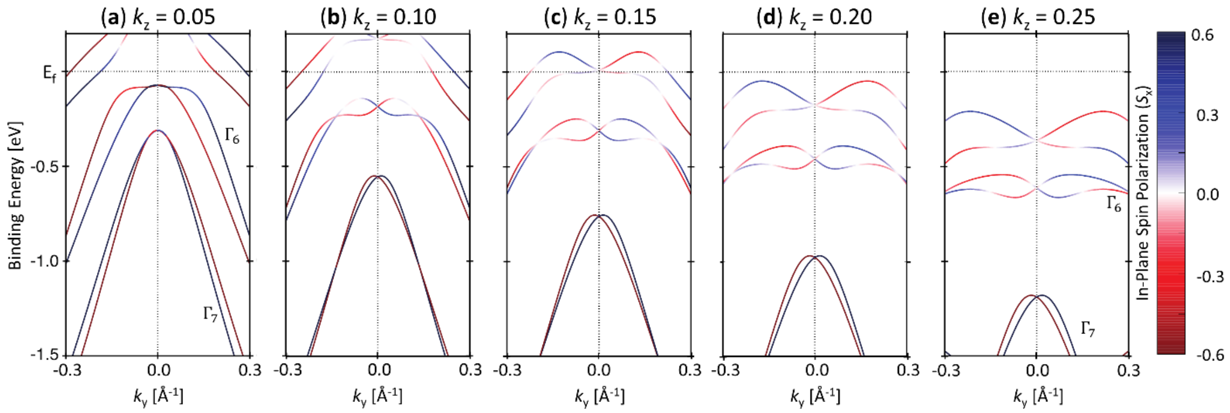

Supplementary Figure 5 | Calculated spin-character of the projected bulk band structure. **a-e**, Calculated spin polarization (S_x) for increasing normalized k_z values between 0.05 and 0.25. Normalized k_z = 0 corresponds to the bulk Γ point and normalized k_z = 0.5 corresponds to the bulk X point. Positive (blue) spin-polarizations correspond to polarization toward positive k_x, negative (red) spin-polarizations correspond to polarization toward negative k_x. Bulk spin polarizations are possible due to the lack of bulk inversion symmetry. Although bulk spin polarizations are predicted, we do not observe them experimentally due to the weak intensity of the bulk bands and the low efficiency of the spin detector.

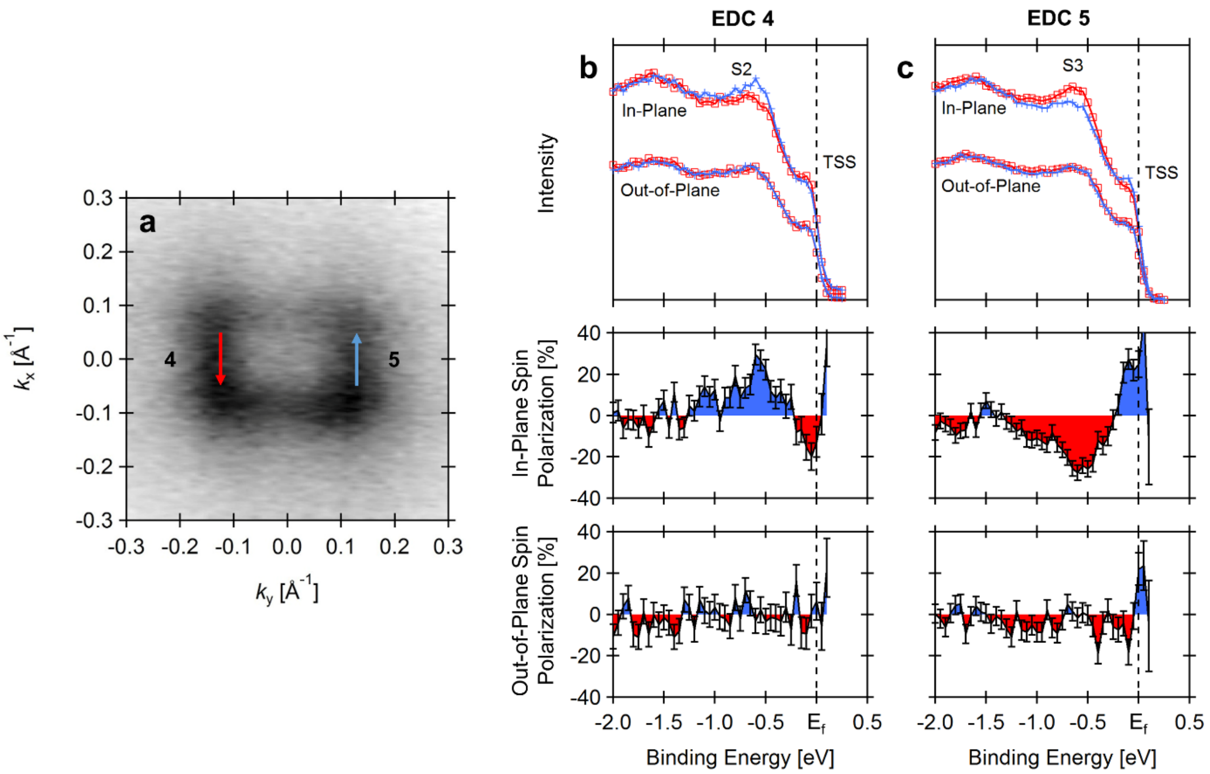

Supplementary Figure 6 | Additional spin-resolved spectra acquired at a photon energy of 18 eV. **a**, Constant binding energy surface with a photon energy of 18 eV highlighting the surface state with linear dispersion and noting the measured spin-polarization orientations. **b-c**, Energy distribution curves and spin polarizations for the noted locations. Measured intensities are shown for the in-plane and out-of-plane spin EDCs. For in-plane spin-polarization, positive (blue) spin-polarizations correspond to polarization toward positive k_x, negative (red) spin-polarizations correspond to polarization toward negative k_x. For out of-plane spin-polarization, positive (blue) spin-polarizations correspond to polarization toward positive k_z, negative (red) spin-polarizations correspond to polarization toward negative k_z. The observed spin-polarization spectra are unchanged compared to those taken at 16 eV.

Supplementary Note 1 | Theory Calculation Alignment

By examining ARPES snapshots for more negative binding energies, the bulk Γ_7 band position can be tracked and aligned with the theory calculations. Supplementary Fig. 2b-2d show such an alignment for snapshots with an incident photon energy of 16 eV, 17 eV, and 18 eV, respectively. Good agreement can be found by shifting the theory calculation Fermi level -0.35 eV (with an approximate error on the order of 0.05 eV). Notably, this alignment deviates more for low photon energies, binding energies far from the Fermi level, and angles far from normal emission due to the divergence between the measured constant photon energy snapshots and the calculated constant k_z bulk band structure projection (a limitation in the typical sudden-approximation free-electron-like final-state photoemission model[1]).

The extracted surface state position and calculated bulk surface projections near the Γ point can be seen in Supplementary Fig. 2a. The surface state position lies such that it connects the envelope of the bulk Γ_6 band projections to the envelope of the bulk Γ_8 band projections. This agrees with the expectation of a TSS which connects these two inverted bands.

Supplementary Note 2 | Rashba-like Surface State Analysis

By examining ARPES snapshots for additional orientations and constant energy surfaces at lower binding energies, the Rashba-like surface state can be seen more clearly. Supplementary Fig. 3a and 3b highlight the split hole, double-arched, appearance expected for a Rashba or mixed Rashba/Dresselhaus surface state. Supplementary Fig. 3c and 3d show the constant energy surface at a binding energy of -0.74 eV. We note the presence of two generally concentric contours that align with the measured spin polarization locations. There is insufficient resolution to confirm that the observed state has the canonical pure-Rashba appearance but the measured data is consistent with the idea of a Rashba-like trivial surface state. Qualitatively, this state has strong parallels to that seen by Liu *et al.*[2] However, as would be expected, quantitatively, a different binding energy maxima is seen due to the difference in surface orientation and elemental components (i.e. PtLuBi and PtGdBi).

Supplementary Note 3 | Topological Surface State Peak Position Extraction and Extrapolation

Surface state peak position was extracted from several photon energies. Multi-peak fitting of individual photon energies was conducted with four or six peaks depending on the number of bulk bands

expected for the k_z value. Voigt peak functions with a linear background were used to identify the peak positions and individual momenta error in order to capture both the theoretical line shape and experimental broadening. Finally, a linear fit, based on peak positions for the TSS at a number of binding energies, was used to extrapolate toward the Dirac point (Fig. 3). The Voigt peak fit errors were incorporated through the linear curve fits. Utilizing these assumptions and neglecting systematic errors, the statistical error, which includes the intersection error, was computed as:

$$\delta E_{Dirac} = \sqrt{(\delta a_1)^2 + \left(\left| b_1 \cdot \frac{a_2 - a_1}{b_1 - b_2} \right| \sqrt{\left(\frac{\sqrt{\delta b_1{}^2 + \delta b_2{}^2}}{|b_1 - b_2|} \right)^2 + \left(\frac{\sqrt{\delta a_1{}^2 + \delta a_2{}^2}}{|a_1 - a_2|} \right)^2 + \left(\frac{\delta b_1}{|b_1|} \right)^2} \right)^2} \quad (1)$$

where $a_1 + \delta a_1$, $b_1 + \delta b_1$, $a_2 + \delta a_2$, and $b_2 + \delta b_2$ are the two sets of linear fit coefficients and their corresponding errors. However, systematic errors in both the experiment, such as imperfect calibration of the energy scale or alignment of the Fermi level, and analysis, such as the exact functional form of the topological state, cannot be numerically quantified well. Consequently, the real error is most likely much larger than the computed value of ±0.02 eV.

Supplementary Note 4 | Additional Spin-ARPES Characterization

Spin ARPES measurements were performed at the I3 beamline of the MAX-IV laboratory, Sweden[3]. This beamline employs a Scienta R4000 hemispherical electron analyzer, configured to output either to a 2D MCP detector for spin-integrated measurements or to a mini-Mott spin detector. The latter permits simultaneous detection of two spin components by measuring the intensity difference between two channeltron detector pairs, one sensitive to in-plane polarization (along the analyzer slit direction) and one sensitive to out-of-plane polarization.

After scaling the channeltrons by known, constant sensitivity factors and subtracting a dark-count background, spin polarization was computed as[4]:

$$Polarization = \frac{I_A - I_B}{S * (I_A + I_B)} \quad (2)$$

where the value of the Sherman function S = 0.15, and I_A and I_B are the counts recorded by the channeltron pair. Error bars are based on counting statistics, and computed as:

$$\delta Polarization = \sqrt{\frac{1}{N * S^2}} \qquad (3)$$

where *N* is the total intensity measured by a given detector pair.

In Supplementary Fig. 6, we show additional spin-resolved spectra acquired at a photon energy of 18 eV. Despite changing the photon energy, the spin texture observed in the main manuscript is unchanged, confirming that the observed spin polarizations originate from the surface bands, rather than the bulk bands.

Supplementary References

1. Hüfner, S. *Photoelectron Spectroscopy*. (Springer Berlin Heidelberg, 2003). doi:10.1007/978-3-662-09280-4
2. Liu, C. *et al.* Metallic surface electronic state in half-Heusler compounds RPtBi (R = Lu, Dy, Gd). *Phys. Rev. B* **83,** 205133 (2011).
3. Berntsen, M. H. *et al.* A spin- and angle-resolving photoelectron spectrometer. *Rev. Sci. Instrum.* **81,** 035104 (2010).
4. Jozwiak, C. *et al.* A high-efficiency spin-resolved photoemission spectrometer combining time-of-flight spectroscopy with exchange-scattering polarimetry. *Rev. Sci. Instrum.* **81,** 053904 (2010).
